# E2112: randomized phase iii trial of endocrine therapy plus entinostat/placebo in patients with hormone receptor-positive advanced breast cancer

**DOI:** 10.1038/s41523-017-0053-3

**Published:** 2018-01-11

**Authors:** Sri Lakshmi Hyndavi Yeruva, Fengmin Zhao, Kathy D. Miller, Amye J. Tevaarwerk, Lynne I. Wagner, Robert J. Gray, Joseph A. Sparano, Roisin M. Connolly

**Affiliations:** 1 Department of Medicine, Howard University Hospital, Washington, DC USA; 2 Dana Farber Cancer Institute, Boston, MA USA; 3 Indiana University Medical Center, Indianapolis, Ind USA; 4 University of Wisconsin, Madison, WSN USA; 5 Wake Forest University Health Services, Winston-Salem, NC USA; 6 Montefiore Medical Center, Albert Einstein College of Medicine, Bronx, NY USA; 7 Johns Hopkins University, Baltimore, MD USA

## Abstract

Endocrine therapies are effective in the treatment of hormone receptor (HR)-positive breast cancer, however, de novo or acquired treatment resistance is a significant clinical problem. A potential mechanism of resistance involves changes in gene expression secondary to epigenetic modifications, which might be reversed with the use of histone deacetylase (HDAC) inhibitors such as entinostat. The ENCORE 301 phase II randomized, placebo-controlled study demonstrated a significant improvement in progression-free survival (PFS) and overall survival (OS), with the addition of entinostat to exemestane in patients with HR-positive advanced breast cancer with disease progression after prior non-steroidal aromatase inhibitor (AI). These results prompted the development of E2112, a phase III registration trial which is investigating entinostat/placebo in combination with exemestane in patients with locally advanced or metastatic breast cancer who have experienced disease progression after a non-steroidal AI. E2112 aims to validate the preclinical and clinical findings supporting the role of HDAC inhibitors in overcoming resistance to endocrine therapy in breast cancer, and ultimately improve outcomes for patients with advanced breast cancer.

## Introduction

Endocrine therapy is an important component of the adjuvant treatment paradigm for the majority of women with hormone receptor (HR)-positive breast cancer, which accounts for approximately two thirds of cases of breast cancer worldwide. Due to both the clinical activity and the benign side effect profile of endocrine agents, they are also a component of standard management for patients with locally advanced or metastatic (advanced) HR-positive breast cancer.^1^ These agents target estrogen signaling which is a key driver of HR-positive breast cancer cell growth, and treatment often involves sequencing of these agents until treatment resistance occurs or visceral crisis prompts a transition to chemotherapy. Several therapeutic options exist and include selective estrogen receptor modulators (e.g., tamoxifen), the aromatase inhibitors (AIs; anastrozole, letrozole, exemestane) and selective estrogen receptor down-regulators (e.g., fulvestrant).

Therapeutic strategies that combine endocrine therapies with targeted agents aim to improve outcomes for patients by overcoming drug resistance. Aberrations in the cell cycle machinery or abnormal signaling via the PI3K/Akt/mTOR intracellular signaling pathway, are proposed mechanisms by which this resistance can occur.^2^ Clinical trials investigating relevant combinations have led to the United States (US) Food and Drug Administration (FDA) and European Medicines Agency (EMA) approval of new treatment combinations for patients with advanced breast cancer in recent years.^3–5^ These therapeutic advances are welcome and will no doubt improve outcomes for many patients with advanced breast cancer. However, ongoing clinical investigation is still required as treatment resistance ultimately develops and patients will require alternative therapeutic strategies.

**Fig. 1:**
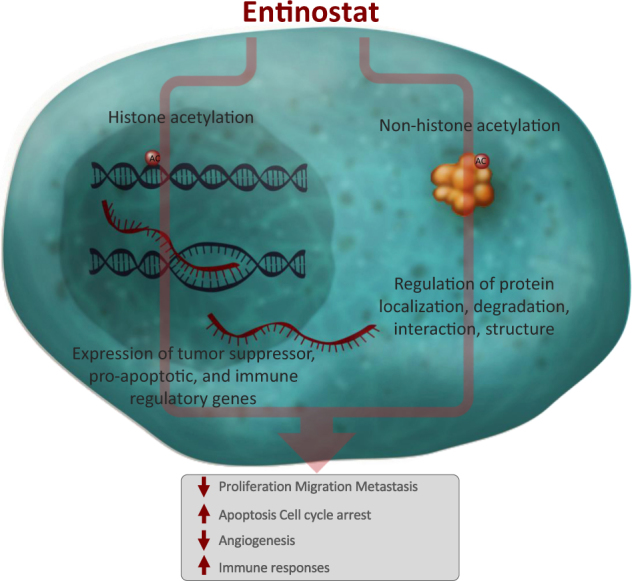
Entinostat mechanism of action. Entinostat impacts cancer not only through its epigenetic actions but also through epigenetic-independent mechanisms by acetylation of non-histone proteins

Alterations in gene expression in breast cancers secondary to epigenetic modifications may also lead to resistance to endocrine therapy.^6^ These epigenetic alterations are frequent in breast cancers and may be modulated with the use of epigenetic modifiers such as histone deacetylase (HDAC) inhibitors. Class-specific inhibitors which target a subset of HDAC enzymes (entinostat and romidepsin) and pan or non-specific HDAC inhibitors (vorinostat, belinostat and panobinostat) have been developed. Currently HDAC inhibitors have been approved only in hematologic malignancies with romidepsin, vorinostat and belinostat approved by the US FDA for treatment of cutaneous or peripheral T-cell lymphoma. Panobinostat is approved in several countries for use in combination with bortezomib and dexamethasone in patients with multiple myeloma. Entinostat, is an oral synthetic benzamide derivative with a long half-life and is administered once per week on an empty stomach (Fig. 1).^7^ It acts by binding to and selectively inhibiting class I and IV HDACs.^8,9^ Histone hyperacetylation results in remodeling of the chromatin structure and allows transcriptional activation of specific genes. Acetylation of non-histone proteins also occurs which can modulate multiple protein properties in the cytoplasm and nucleus of the cancer cell.^10^ These epigenetic-dependent and epigenetic-independent actions of HDAC inhibitors ultimately result in reduced tumor growth through inhibition of cell proliferation and metastasis, terminal differentiation, and apoptosis.^11^ Entinostat is not yet approved by regulatory agencies for any indication. However, both clinical and preclinical evidence support a potential role of entinostat in treating hormone-resistant breast cancer.

**Fig. 2:**
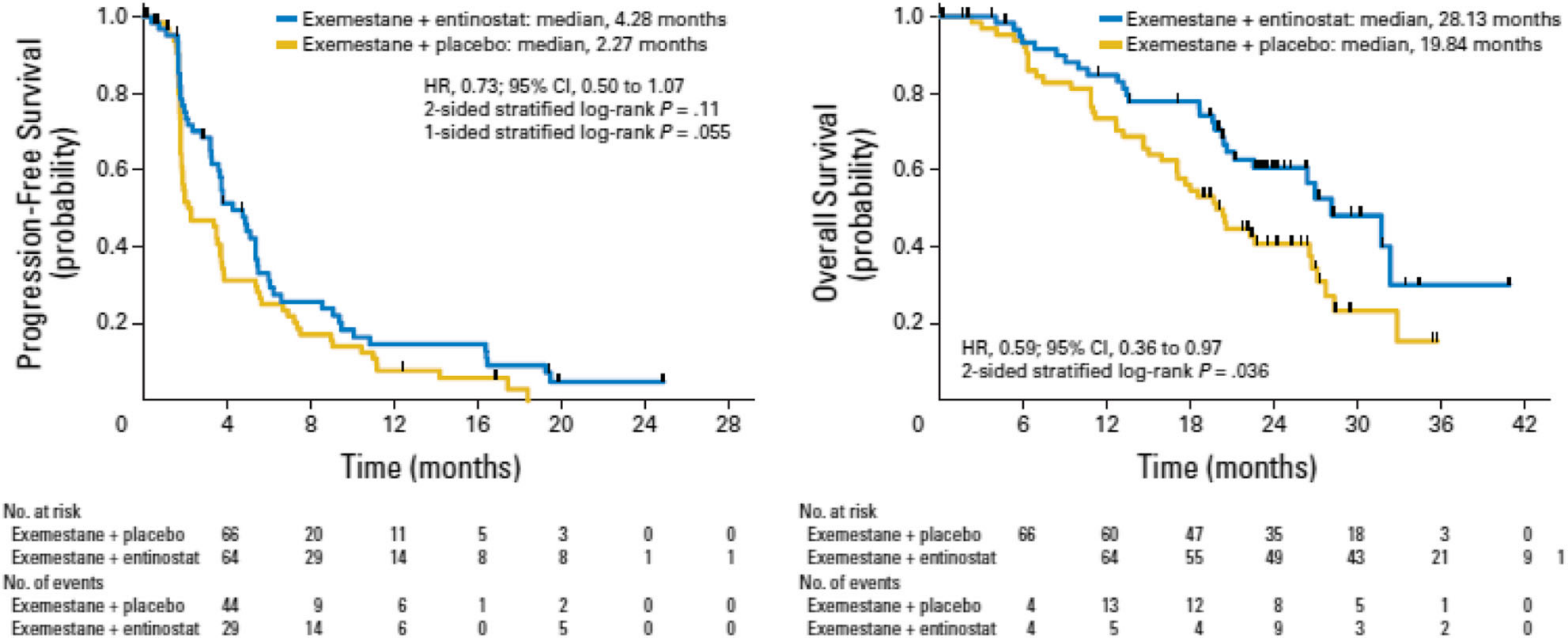
Kaplan–Meier estimates of progression-free and overall survival in the ENCORE301 trial. Adapted with permission from Yardley et al. JCO 2013

ENCORE 301 was a phase II randomized, placebo-controlled study which evaluated the addition of entinostat to the steroidal AI exemestane in patients with HR-positive advanced breast cancer with disease progression after prior non-steroidal AI. The study demonstrated a significant improvement in progression-free survival which was the primary study endpoint (PFS, hazard ratio [HR] 0.73; 95% CI, 0.50 to 1.07; *p* = 0.06) (Fig. 2) and also overall survival (OS, HR, 0.59; 95% CI, 0.36 to 0.97; *p* = 0.036) (Fig. 2). The combination was well tolerated, with neutropenia (13%) and fatigue (11%) being the most frequent grade 3 or 4 toxicities in entinostat-treated patients.^12^ Finally, in an attempt to identify a predictive biomarker of response to this combination, protein acetylation in peripheral blood mononuclear cells (PBMCs) at baseline and two weeks after commencement of entinostat and endocrine therapy was evaluated in a subset of patients in the ENCORE301 study.^12^ The median PFS was significantly longer in those patients with protein lysine hyperacetylation versus those who did not exhibit same (8.5 versus 2.7 months, HR 0.32, 95% CI 0.13–0.79).

In preclinical models, the combination of entinostat and AI therapy significantly reduced tumor volume in letrozole-resistant mouse xenograft models when compared to treatment with either agent alone.^13^ Mechanistic studies revealed that the HDAC inhibitor increased expression of *ER* and aromatase activity but downregulated *HER2*, and also phosphorylated HER2/MAPK and AKT. Thus posttranslational and transcriptional modulation of HER2 rather than reversal of epigenetic silencing may be the primary mechanism through which entinostat was able to overcome treatment resistance.^13^ Other mechanisms by which HDAC inhibitors may improve outcomes in breast cancer are under investigation and include their impact on epithelial-mesenchymal transition (EMT) and modulation of the tumor microenvironment.^14–16^ These mechanisms may explain the large improvement in OS observed in the phase II ENCORE301 study in the setting of a modest PFS improvement, as has been observed with immune therapies in other tumor types.^17^

The promising results of the ENCORE301 study led to FDA designation of entinostat as a Breakthrough Therapy for treatment of HR-positive advanced breast cancer when added to exemestane in postmenopausal women whose disease has progressed after nonsteroidal AI therapy.^18^ A phase III registration trial has thus been developed with input from the FDA (E2112, ClinicalTrials.gov identifier: NCT02115282). E2112 is investigating exemestane in combination with entinostat/placebo in patients with locally advanced or metastatic breast cancer who have experienced disease progression after a non-steroidal AI and is the focus of this review.

## Summary of trial design

**Fig. 3:**
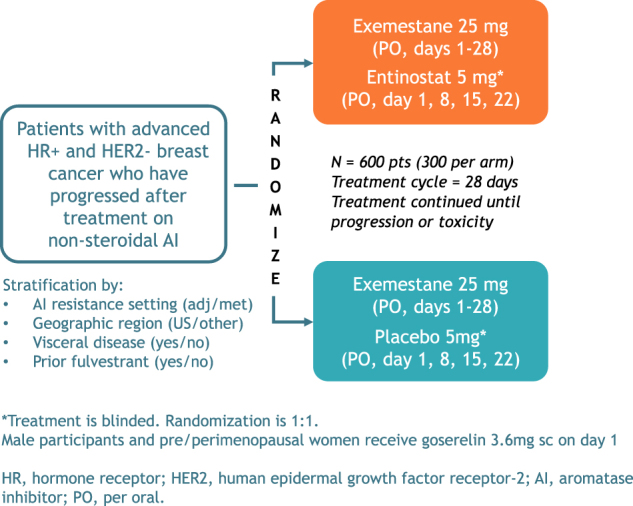
E2112 study schema

E2112 is an international randomized double blind placebo-controlled phase III trial of endocrine therapy (exemestane) plus entinostat/placebo in men and premenopausal and postmenopausal women with HR-positive and HER2-negative locally advanced or metastatic breast cancer who have experienced disease progression after a non-steroidal AI (in advanced setting, or relapse while on or within ≤ 12 months of the end of adjuvant non-steroidal AI). Table 1 highlights key features of the trial including eligibility criteria. Figure 3 outlines the study schema. Stratification factors include setting in which patient developed resistance to prior non-steroidal AI (adjuvant/metastatic), geographic region, presence of visceral disease and prior fulvestrant use.

**Table 1:** Trial at a glance

Rationale	- Preclinical studies suggest that the HDAC inhibitor entinostat can overcome resistance to non-steroidal AI therapy in breast cancer.
	- Phase II ENCORE301 randomized trial reported an 8 month OS benefit for addition of entinostat to exemestane.
Hypothesis	The addition of the HDAC inhibitor entinostat to exemestane will improve PFS and/or OS in patients with HR-positive, HER2-negative advanced breast cancer with disease progression after prior non-steroidal AI.
Primary endpoints	PFS and/or OS
Secondary endpoints	- Safety and tolerability
	- Objective response rate
	- Change in acetylation status in PBMCs
	- Time to treatment deterioration
	- Health-related quality of life
	- Specific symptoms associated with entinostat
	- Adherence to protocol therapy
	- Pharmacokinetics
Exploratory endpoints	Patient ratings of AEs using PRO-CTCAE items
Integrated biomarkers	Change in protein lysine acetylation in PBMCs collected at baseline and 15 days after initiation of study therapy
Sample size	-Randomized double blind placebo-controlled phase III trial with 1:1 randomization
	-Sample size of 600 pts provides adequate power for OS endpoint
	-One-sided Type 1 error of 0.025 split; 0.001 for the PFS test and 0.024 for the OS test
	-PFS tested in first 360 pts: 88.5% power to detect 42% reduction in the hazard of PFS failure (median PFS 4.1 to 7.1 months)
	-OS tested in all 600 pts: 80% power to detect 25% reduction in the hazard of death
	(median OS 22 to 29.3 months)
Patient population	-Premenopausal and postmenopausal women, and men ( ≥ 18 yrs)
	-Locally advanced/metastatic HR-positive, HER2 negative breast cancer
	-Disease progression after non-steroidal AI in advanced setting, or relapse on or within ≤ 12 months of end of adjuvant non-steroidal AI
	-Measurable or evaluable (approx.. 20% cap) disease
	-Prior fulvestrant, everolimus, CDK inhibitor, one prior chemotherapy regimen for metastatic disease permitted
	-ECOG performance status 0–1
	-No history of CNS metastases

*AEs* adverse events, *AI* aromatase inhibitor, *CNS* central nervous system, *CDK* cyclin-dependent kinase, *HER2* human epidermal growth factor receptor-2, *HDAC* histone deacetylase, *HR* hormone receptor, *OS* overall survival, *PBMCs* peripheral blood mononuclear cells, *PFS* progression-free survival

All participants sign a written informed consent approved by the Central Institutional Review Board (IRB) of the National Cancer Institute (NCI) or by the participating institution’s local IRB. Participating institutions follow local regulatory policies per Good Clinical Practice. The trial was designed in consultation with the FDA and is being conducted by ECOG-ACRIN under the sponsorship of the NCI. Syndax Pharmaceuticals Inc. is supporting the trial under a Cooperative Research and Development Agreement with the NCI and a separate agreement with ECOG-ACRIN. Screening and patient enrollment was initiated to the study in March 2014 across the NCI’s National Clinical Trials Network (NCTN).

The primary objective of E2112 is to determine whether the addition of entinostat to exemestane improves PFS and/or OS in patients with HR-positive and HER2-negative locally advanced or metastatic breast cancer who have progressed on prior non-steroidal AI. A sample size of 600 patients (300 per arm) is required to provide adequate power for the OS endpoint. Both PFS and OS are primary endpoints, and the study is designed to show an improvement in either PFS or OS. The one-sided Type I error of 0.025 is split between two hypotheses tests to control the overall Type I error rate for the trial. The primary analysis of PFS will be performed using a stratified log-rank test, with one-sided Type I error of 0.1%, stratifying on randomization stratification factors. The primary analysis of OS will be performed using a stratified log-rank test, with one-sided Type I error of 2.4%, also stratifying on randomization stratification factors. PFS is tested in the first 360 patients and the study provides 88.5% power to detect 42% reduction in the hazard of PFS failure (median PFS, 4.1 to 7.1 months). OS is tested in all 600 patients with 80% power to detect 25% reduction in the hazard of death (median OS, 22 to 29.3 months). An interim futility analysis plan for PFS is incorporated in the study design, as well as an interim efficacy/futility analysis plan for OS. Additional details regarding secondary/exploratory objectives and statistical analysis plan are provided in Table 1. Archival tumor samples and blood samples will be collected to explore potential biomarkers of therapeutic efficacy.

Patients are randomized in a 1:1 ratio to receive either entinostat plus exemestane or exemestane plus placebo. Patients receive exemestane 25 mg by mouth from day 1 to 28 and entinostat/placebo 5 mg by mouth on days 1, 8, 15 and 22. Treatment cycles are repeated every 28 days until disease progression or development of unacceptable toxicities. Male participants and pre/perimenopausal women also receive goserelin 3.6 mg subcutaneously monthly.

Tumor response and progression will be evaluated in this study using the revised Response Evaluation Criteria in Solid Tumors (RECIST) guideline (version 1.1). Tumor response and progression will be defined by central review. Adverse events will be graded using the NCI Common Terminology Criteria for Adverse Events (CTCAE) version 4.0. The patient-reported outcomes assessment for this protocol will measure overall HRQL, treatment-related toxicities, breast cancer-specific symptoms, and adherence to protocol therapy (PROMIS Fatigue, FAACT, FACIT-D, FACT-G, FBSI, Morisky Medication Adherence Scale). Blood sampling for lysine acetylation, a biomarker of entinostat activity, takes place at cycle 1 day 1, and cycle 1 day 15. Blood sampling for pharmacokinetic analyses is optional and takes place at cycle 1 day 1, cycle 1 day 15 and prior to cycle 2.

## Discussion

The phase III E2112 trial was developed with input from the FDA after receipt of Breakthrough Therapy Designation for entinostat when used in combination with exemestane in HR-positive advanced breast cancer.^18^ The trial aims to confirm the results of the ENCORE 301 study which demonstrated a significant improvement in PFS and OS (Fig. 2) with the addition of entinostat to exemestane in patients with HR-positive advanced breast cancer with disease progression after prior non-steroidal AI.^12^ The significant improvement in OS of approximately 8 months in entinostat-treated patients was an unexpected, albeit exploratory, result and has not been observed with any treatment combination in this setting to date. It has been hypothesized that an impact of entinostat on epithelial-mesenchymal transition (EMT) and modulation of the tumor microenvironment may explain the large improvement in OS observed in the ENCORE301 study in the setting of a modest PFS improvement.^14–16^ Indeed a significant reduction in granulocytic myeloid-derived suppressor cells (MDSCs) and monocytic MDSCs has been reported in PBMCs in a retrospective analysis of samples from entinostat-treated patients in ENCORE301.^19^

Treatment options for patients with HR-positive advanced breast cancer should ideally prolong survival, improve symptom control and enhance quality of life. Agents and treatment combinations thus with a favorable side effect profile and a convenient treatment schedule may be preferable. The first approval of an endocrine therapy combined with an additional therapy in this space was based on data from the BOLERO-2 trial.^3^ The combination of exemestane and everolimus (an mTOR inhibitor) resulted in a 4 month PFS advantage when compared to exemestane plus placebo in patients who had previously received a non-steroidal AI. Early results from the phase II PrECOG0102 trial also indicate a PFS advantage for the addition of everolimus to fulvestrant which requires confirmation.^20^ More recently, the cyclin-dependent kinase (CDK) 4/6 inhibitor palbociclib and letrozole combination was approved for patients with HR-positive advanced breast cancer as a front-line therapy after a significant improvement in PFS was observed in both the PALOMA-1 and PALOMA-2 trials for the combination versus letrozole alone.^5,21^ PALOMA-3 also indicated a PFS advantage for the combination of palbociclib with fulvestrant in later-line therapy.^4^ Similar results have been observed with the CDK inhibitor ribociclib (MONALEESA-2 trial), which is also now FDA approved in the 1st line setting in combination with an AI.^22^ Abemaciclib with or without fulvestrant is currently pending regulatory approval based on MONARCH2 and other trial data.^23^

If E2112 reports positive results, it may lead to FDA approval for this regimen. With this explosion of new treatment options for patients with HR-positive advanced breast cancer, we must consider where the combination of exemestane and entinostat might fit in the treatment paradigm. Based on the study eligibility criteria, it is anticipated that patients enrolling in E2112 may have received a prior CDK inhibitor, and may or may not have received one prior chemotherapy regimen for metastatic disease. Thus, the combination could realistically be prescribed in either the 1st line setting, or indeed in later lines after CDK inhibitor use; as long as a patient has experienced disease progression after an AI. As E2112 is enrolling patients with any menopausal status and also patients with male breast cancer, the study results will be applicable to these patient subgroups. Reassuringly, the combination being investigated in E2112 has been shown in prior studies to be a safe and well tolerated oral regimen.^12^ The ENCORE301 trial reported the most frequent adverse events (predominantly grade 1/2) in entinostat-treated patients to be fatigue (11% grade 3), weight loss, gastro-intestinal toxicity, hematologic toxicity (e.g., 13% grade 3 neutropenia), dyspnea, and peripheral edema.^12^ Therefore, the decision to prescribe this treatment combination if approved, may depend on the adverse event profile when compared to other available combinations, as well as the magnitude of clinical benefit observed.

Strengths of the E2112 study include its randomized placebo-controlled design as well as the eligibility criteria which closely parallels that of the phase II ENCORE301 trial. An amendment early in study conduct did, however, alter the eligibility to include premenopausal patients as well as use of prior fulvestrant. E2112 is also exploring a promising biomarker in patients treated with exemestane and entinostat. In a preplanned secondary analysis of ENCORE301, the median PFS was significantly longer in those patients with protein lysine hyperacetylation in PBMCs two weeks after commencement of entinostat and endocrine therapy versus those who did not (8.5 versus 2.7 months).^12^ If this is confirmed as a prognostic biomarker in E2112, we may in the future be able to personalize the treatment approach for patients with HR-positive advanced breast cancer receiving this combination. Additional studies to identify prognostic and predictive biomarkers will be undertaken using valuable archival tumor specimens and prospectively collected blood samples. The incorporation of patient-reported outcome measures is also a strength of the study. A limitation of the study, and a challenge for many co-operative group studies, is that the provision of archival tumor specimens was not mandatory for study participation, and research tumor biopsies are not being collected.

As we continue along this path to providing better treatment options for patients, strong collaboration between clinical investigators, basic scientists, regulatory bodies and industry partners is essential to enable timely drug and biomarker development. This paradigm will maximize the important goal of a personalized approach to care for patients which avoids both undertreatment and overtreatment, and ultimately results in long-term control of breast cancer.
